# Relationship Between the Degree of Diabetic Retinopathy and Serum Fractalkine (CX3CL1) in Patients with Type 2 Diabetes: A Single-Center Cross-Sectional Study

**DOI:** 10.3390/medicina62020312

**Published:** 2026-02-02

**Authors:** Ozgur Yilmaz, Mehmet Erdogan, Murvet Algemi, Ibrahim Kocak, Sengul Aydin Yoldemir, Murat Akarsu

**Affiliations:** 1 Department of Internal Medicine, Kanuni Sultan Suleyman Training and Research Hospital, Istanbul 34303, Turkey; 2 Department of Ophthalmology, Kanuni Sultan Suleyman Training and Research Hospital, Istanbul 34303, Turkey; 3 Department of Biochemistry, Kanuni Sultan Suleyman Training and Research Hospital, Istanbul 34303, Turkey

**Keywords:** diabetic retinopathy, fractalkine, Type 2 diabetes, microvascular complications, cross-sectional study

## Abstract

*Background and Objectives:* Diabetic retinopathy (DR) is a leading microvascular complication of type 2 diabetes (T2D). Fractalkine (CX3CL1), a chemokine involved in inflammation, angiogenesis, and microglial activation, may play a role in DR pathogenesis. This study investigated the association between serum fractalkine levels, the presence of DR, and disease severity. *Materials and Methods:* In this cross-sectional study, 140 adults with T2D were classified as non-DR (n = 32) or DR (n = 108) according to ICDR and ETDRS criteria; DR cases were further categorized into NPDR (n = 76) and PDR (n = 32), with NPDR staged as mild, moderate, or severe. Serum fractalkine concentrations were measured using ELISA. *Results:* Serum fractalkine levels were significantly higher in patients with DR than in those without retinopathy (0.7 vs. 0.4 ng/mL, *p* < 0.001). Within NPDR stages, fractalkine levels were highest in severe NPDR (*p* = 0.004). No significant fractalkine difference was found between NPDR and PDR groups. In multivariable analysis, serum fractalkine (OR 10.2; 95% CI 1.2–89.6; *p* = 0.036) remained independently associated with the presence of DR. For identifying DR, fractalkine yielded an AUC of 0.736; the optimal cut-off of 0.455 ng/mL provided 81.5% sensitivity and 56.3% specificity. In distinguishing severe NPDR, fractalkine demonstrated strong diagnostic performance (AUC = 0.784), with a cut-off of 0.720 ng/mL yielding 100% sensitivity and 61.9% specificity. *Conclusions:* Serum fractalkine is significantly associated with both the presence and severity of DR and remains independently associated with retinopathy after adjustment for traditional risk markers. Serum fractalkine may offer complementary systemic information in automated and AI-based retinal screening. These findings are exploratory and hypothesis-generating, and prospective studies are required to determine the clinical relevance of serum fractalkine in DR.

## 1. Introduction

Diabetic retinopathy (DR) is one of the most serious microvascular complications of diabetes mellitus and a leading cause of vision loss in adults worldwide [1]. The World Health Organization estimates that by 2045, there will be 700 million people living with diabetes globally, of whom approximately 30–40% will develop DR [2]. The pathogenesis of DR involves complex metabolic, molecular, and cellular changes driven by chronic hyperglycemia [3].

Pathological changes in the retinal microvascular system constitute the basic features of DR. These include pericyte loss, basement membrane thickening, disruption of the blood–retinal barrier, capillary occlusion, and retinal ischemia [4,5]. Complications that arise in later stages—such as proliferative changes, vitreous hemorrhage, tractional retinal detachment, and diabetic macular edema—can lead to irreversible vision loss [6]. The progressive nature of DR necessitates early diagnosis and the development of effective treatment strategies [7].

In recent years, the role of inflammatory processes in the pathogenesis of DR has been increasingly understood [8]. Chronic hyperglycemia activates the inflammatory cascade by increasing oxidative stress in the retinal tissue and leads to the release of various cytokines, chemokines and growth factors. Among these molecules, vascular endothelial growth factor (VEGF), tumor necrosis factor-alpha and various chemokines play important roles [9,10].

Fractalkine (CX3CL1) is a bifunctional molecule belonging to the CX3C chemokine family that is structurally different from other chemokines [11]. Fractalkine, which exists in both soluble and membrane-bound forms, plays a critical role in various pathophysiological processes such as inflammation, angiogenesis, neurodegeneration, and immune modulation. Fractalkine, which is expressed especially by retinal ganglion cells in the retina, binds to the CX3C chemokine receptor 1 (CX3CR1) on microglia and plays an important regulatory role in maintaining neurovascular unit homeostasis [12].

Recent studies on the role of the fractalkine-CX3CR1 axis in the pathogenesis of DR have revealed the complex and multifaceted functions of this system [13]. Increased fractalkine expression in diabetic retina can exert both neuroprotective and proinflammatory effects by modulating microglial activation. Defects in fractalkine signaling can lead to exacerbation of retinal inflammation, increased vascular damage, and neuronal degeneration. Experimental studies have shown that fractalkine administration can reduce retinal inflammation and prevent neurovascular damage [14,15].

In clinical studies, it has been suggested that fractalkine levels correlate with the severity of DR and may be a potential biomarker for predicting disease progression. It has been reported that fractalkine levels are increased in vitreous samples of patients with proliferative DR (PDR). However, studies systematically examining the relationship between serum fractalkine levels and DR stages are limited [16,17].

In recent years, advances in automated retinal screening systems and artificial intelligence-based algorithms have enabled large-scale and standardized detection of DR, particularly in primary care and underserved settings. These technologies have shifted clinical focus toward earlier identification, risk stratification, and monitoring of disease progression. In this context, the identification of complementary systemic biomarkers may provide additional value by supporting clinical decision-making and enhancing the interpretation of automated screening outputs. Recent studies comparing autonomous artificial intelligence systems with teleophthalmology-based screening models further highlight the need for clinically relevant biomarkers that can be integrated into contemporary DR screening strategies [18,19].

The primary objective of this study was to evaluate the association between serum fractalkine levels and DR severity across clinically defined disease stages. The secondary objectives were to examine the relationships between serum fractalkine levels and key clinical markers, including diabetes duration and glycated hemoglobin (HbA1c), and to explore whether circulating fractalkine levels reflect systemic inflammatory activity in the context of DR.

## 2. Materials and Methods

### 2.1. Study Design and Setting

This single-center, cross-sectional study was conducted in the Department of Internal Medicine at Kanuni Sultan Süleyman Training and Research Hospital, in accordance with the principles of the Declaration of Helsinki. An a priori power analysis (Cohen’s d = 0.5, α = 0.05, power = 80%) indicated a minimum sample size of 120, which was exceeded by the final cohort of 140 patients. A total of 140 adults diagnosed with type 2 diabetes (T2D), based on the American Diabetes Association (ADA) 2024 diagnostic criteria [20], and presenting to the hospital’s internal medicine outpatient clinics were included. Participants were classified into two primary groups according to the presence of DR: the non-DR group (n = 32) and the DR group (n = 108). Patients with DR were further categorized by disease severity into non-proliferative DR (NPDR) (n = 76) and PDR (*n* = 32). The NPDR group was additionally subclassified into mild (n = 23), moderate (*n* = 40), and severe (*n* = 13) stages according to standardized ophthalmologic assessment criteria. This study was reported in accordance with the Strengthening the Reporting of Observational Studies in Epidemiology (STROBE: Supplementary Material) statement [21,22].

### 2.2. Diabetic Retinopathy Assessment Method

The diagnosis and classification of DR were made based on the International Diabetic Retinopathy Severity Scale (ICDR) and the Early Treatment of Diabetic Retinopathy Study (ETDRS) criteria by the same retina specialist (with at least 5 years of experience) who was unaware of the clinical features of the patients. All participants underwent a comprehensive ophthalmologic evaluation, including dilated fundus examination using a +90 diopter lens, 45-degree color fundus photography (Topcon TRC-50DX, Topcon Corporation, Tokyo, Japan), fluorescein angiography (Heidelberg Spectralis HRA-2, Heidelberg Engineering GmbH, Heidelberg, Germany), and spectral-domain optical coherence tomography (OCT) imaging (Heidelberg Spectralis, Heidelberg Engineering GmbH, Heidelberg, Germany). The patients were classified according to the ICDR criteria: mild NPDR (microaneurysms only), moderate NPDR (microaneurysms + retinal hemorrhage + hard exudate), severe NPDR (4-2-1 rule positivity: more than 20 retinal hemorrhages, venous beading, or overt intraretinal microvascular abnormalities), and PDR (retinal/optic disk neovascularization). The criteria for the diagnosis of diabetic macular edema were central macular thickness > 250 µm on Spectralis OCT and/or leakage detected on fluorescein angiography. Disagreements and diagnostic disagreements were resolved by a second ophthalmologist [23,24].

### 2.3. Exclusion Criteria

Primary exclusion criteria included; type 1 diabetes mellitus, diabetic ketoacidosis, malignancies, severe liver disease, acute or end stage renal failure, active infections, chronic inflammatory or autoimmune disorders, and chronic pulmonary diseases Additionally, patients with thyroid disorders, neurological diseases, a history of ocular surgery or retinal disease, prior anti-VEGF treatment or laser photocoagulation, organ transplantation, malnutrition, systemic steroid use, as well as pregnant or breastfeeding individuals, were also excluded.

### 2.4. Data Collection

Sociodemographic data, including age, gender, family history of diabetes, and duration of diabetes mellitus, were obtained through patient interviews and verified using the hospital’s electronic medical records. Body weight and height were measured using standardized equipment, and body mass index (BMI) was calculated as weight divided by height squared (kg/m^2^). Blood pressure was recorded from the right arm following a 15 min rest period. All patients underwent systematic screening for DR as part of the study protocol. Current medications and major comorbid conditions—including diabetic nephropathy, neuropathy, cardiovascular and cerebrovascular disease, peripheral vascular disease, dyslipidemia, hypertension, and metabolic syndrome were documented and cross-checked with the electronic medical record system.

### 2.5. Blood Sampling and Biochemical Analysis

Fasting venous blood samples were obtained between 8:00 and 10:00 a.m. following a 10–12 h overnight fast. Blood was drawn from the antecubital fossa into plain, anticoagulant-free tubes for serum analyses and into appropriate tubes for plasma collection when required. All laboratory personnel performing the assays were blinded to patient group allocation to minimize measurement bias. Samples were centrifuged at 4 °C for 20 min at 2000 rpm, and biochemical analyses were conducted immediately on freshly separated serum samples. Fasting plasma glucose, creatinine, alanine aminotransferase (ALT), albumin, and the lipid profile (total cholesterol, triglycerides, HDL-C, and LDL-C) were measured using enzymatic colorimetric methods on the Roche Cobas 8000 c702 analyzer (Roche Diagnostics, Mannheim, Germany). C-reactive protein (CRP) levels were assessed by an immunoturbidimetric method on the same analyzer. HbA1c was determined using high-performance liquid chromatography with the ARKRAY/ADAMS HA-8180V system (ARKRAY Inc., Kyoto, Japan). Serum and plasma aliquots designated for additional analyses were promptly frozen and stored at −80 °C until further evaluation.

### 2.6. Measurement of Serum Fractalkine (CX3CL1) Levels

Venous blood samples were collected for fractalkine and centrifuged, and the sera were separated into Eppendorf tubes and stored at −80 °C until analysis. A commercial sandwich enzyme-linked immunosorbent assay (ELISA) kit (E-EL-H0044, Elabscience, Wuhan, China) was used to measure human fractalkine concentrations. Experiments were performed according to the manufacturer’s protocol, as follows: (1) 100 μL of standard/sample was added to micro ELISA plates and incubated for 90 min at 37 °C; (2) biotinylated detection antibody (1:100 dilution) was added and incubated for 60 min; (3) horseradish peroxidase-conjugate (1:100 dilution) was added after 3× washing and incubated for 30 min; (4) tetramethylbenzidine substrate was added after 5× washing and incubation in the dark for 15 min to initiate the color reaction; (5) stop solution was added and optical density (OD) was measured at 450 nm. A calibration curve was generated using a four-parameter logistic (4-PL) model within the analytical range of 0.16–10 ng/mL, with an assay sensitivity of 0.10 ng/mL. Plasma/serum samples were anticoagulated with EDTA and centrifuged at 1000× *g*, and samples exceeding the linear range were analyzed after stepwise dilution (100–100,000-fold). All measurements were performed in duplicate, and the repeatability of the assay was supported by intra-assay and inter-assay coefficients of variation (CV) of <10%. According to the manufacturer, no cross-reactivity with structurally related proteins was observed. Additional analytical validation data provided by the manufacturer demonstrated excellent linearity (r > 0.99), acceptable recovery (90–110%), and inter-assay precision < 12%. Independent in-house validation was not performed; however, duplicate measurements were used to ensure reproducibility.

### 2.7. Outcomes

The primary outcome of this study was to assess serum fractalkine levels across patients without retinopathy and those with varying stages of DR. Secondary outcomes included evaluating the relationship between serum fractalkine levels and retinopathy severity, as well as assessing diagnostic performance through receiver operating characteristics (ROC) analysis and its incremental value beyond established clinical risk factors.

### 2.8. Statistical Analysis

Statistical analyses were performed using IBM SPSS Statistics version 25.0 (IBM Corporation, Chicago, IL, USA) and R software version 3.6.1. The distribution of continuous variables was assessed using the Shapiro–Wilk test. For normally distributed data, comparisons between two groups were conducted using Student’s *t*-test, and multiple-group comparisons were performed using one-way ANOVA followed by Tukey’s post hoc test. For non-normally distributed variables, the Mann–Whitney U test was applied for two-group comparisons, while the Kruskal–Wallis test with Dunn’s post hoc correction was used for comparisons across more than two groups. Correlations between serum fractalkine concentrations and clinical or biochemical parameters, including retinopathy severity, were evaluated using Pearson or Spearman correlation coefficients according to data distribution. Candidate variables were selected a priori based on established pathophysiological domains relevant to DR, including diabetes duration, glycemic control, renal function, and systemic inflammation. Variables demonstrating statistical significance in univariate analyses, together with those deemed clinically relevant, were subsequently entered into multivariable logistic regression models using a forward likelihood ratio approach, with the aim of identifying independent associations while reducing the risk of model overfitting. Diagnostic performance was examined using ROC curve analysis. The area under the curve (AUC) was calculated, and optimal cut-off values were determined using the Youden index, followed by assessments of sensitivity, specificity, and predictive values. Results are presented as mean ± standard deviation for normally distributed variables and median (interquartile range) for non-normally distributed variables. A two-tailed *p* value < 0.05 was considered statistically significant.

## 3. Results

**Figure 1:**
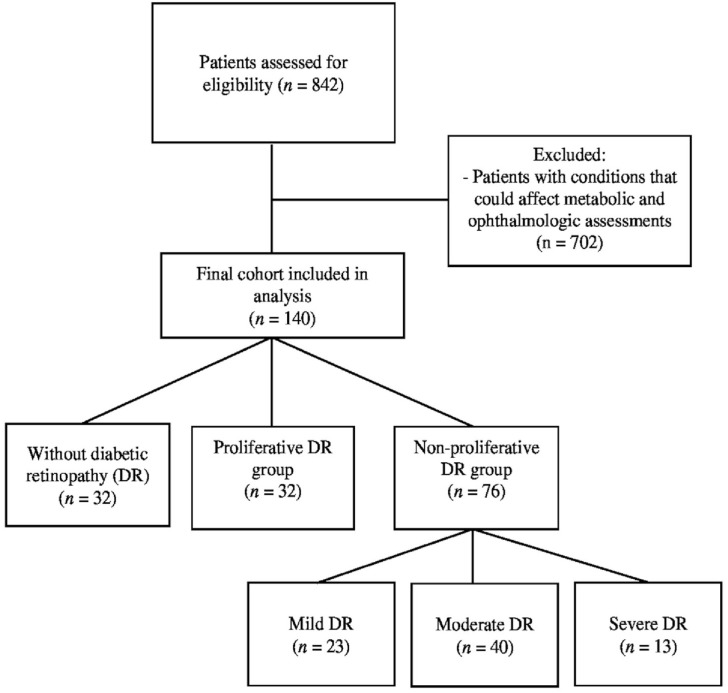
Flow diagram of the study.

A total of 140 T2D patients were included in this study. 32 (22.9%) of the participants were individuals without DRP and 108 (77.1%) were diagnosed with DR. The DR group was divided into two subgroups as PDR and NPDR, with 32 (29.6%) PDR patients and 76 (70.4%) NPDR patients. The NPDR group was further divided into three subgroups as mild (30.3%, *n* = 23), moderate (52.6%, *n* = 40) and severe (17.1%, *n* = 13). A flow diagram summarizing patient selection and exclusions is provided in Figure 1.

The mean age of the participants was 56.8 ± 9 years, and the duration of diabetes was 11.4 ± 8 years. The male/female ratio was 49.3%/50.7%, and the mean BMI was 29.2 ± 4.5 kg/m^2^. Hypertension was present in 32.9% of the participants. Among patients with DR, 42.6% used oral agents alone, 6.5% used insulin alone, and 42.2% used a combination. Demographic and clinical data were compared between groups with and without retinopathy, and detailed information is presented in Table 1.

Serum fractalkine concentrations were significantly higher in patients with DR compared with those without retinopathy (median 0.7 (0.42–0.85) vs. 0.4 (0.40–0.95) ng/mL, *p* < 0.001). Glycemic and renal parameters, including fasting blood glucose, HbA1c, urea, creatinine, WBC count and urinary microalbumin indices, were also elevated in the retinopathy group. Detailed comparisons are presented in Table 2.

According to the logistic regression analysis (Table 3), several variables were associated with the presence of DR in univariate models, including diabetes duration, serum fractalkine levels, fasting blood glucose, HbA1c, serum urea, creatinine, and leukocyte count. In multivariable logistic regression analysis, serum fractalkine remained independently associated with DR (OR 10.2), indicating a positive association; however, the wide 95% confidence interval (1.2–89.6) reflects substantial uncertainty in the estimated effect size, likely related to sample size and variability. Notably, beyond serum fractalkine, both HbA1c and serum creatinine remained independently associated with DR in the multivariable model. In contrast, UACR did not demonstrate significant discriminative ability based on AUC analysis, either when evaluated alone or within multivariable ROC models.

To further examine the association between serum fractalkine and DR in relation to glycemic status, HbA1c-stratified logistic regression analyses were performed. Participants were stratified according to HbA1c levels (<7% and ≥7%), and both univariate and multivariable regression models were constructed for each subgroup. In these analyses, serum fractalkine was not significantly associated with DR among individuals with HbA1c < 7%. In contrast, among individuals with HbA1c ≥ 7%, serum fractalkine remained significantly associated with DR after multivariable adjustment (Table 4 and Table 5).

Serum fractalkine levels were not significantly correlated with demographic characteristics, glycemic indices, renal parameters, inflammatory markers, or lipid profile components.

When PDR and NPDR groups were compared, serum fractalkine concentrations were numerically lower in the proliferative group; however, the overall difference between disease stages was not clinically meaningful (median 0.5 (0.38–1.03) vs. 0.7 ng/mL (0.44–0.83), *p* = 0.274). Likewise, no significant differences were observed in metabolic, renal, inflammatory, lipid, or hematologic parameters (Table 6).

Among patients with NPDR, serum fractalkine concentrations were significantly higher in the severe subgroup compared with the mild and moderate subgroups (median 0.83 (0.45–0.93) vs. 0.68 (0.41–0.79) and 0.63 (0.46–0.91) ng/mL, respectively), whereas no statistically meaningful difference was observed between the mild and moderate subgroups. The urine albumin-to-creatinine ratio (UACR) was also significantly higher in the severe and moderate groups relative to the mild group. No other laboratory parameters differed significantly across NPDR severity categories (Table 7).

**Figure 2:**
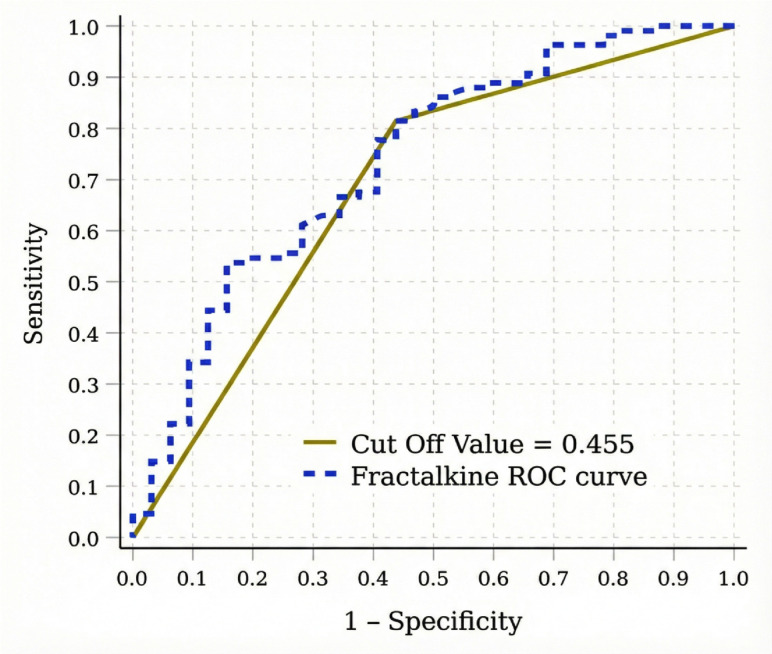
ROC curve of serum fractalkine levels for distinguishing patients with and without diabetic retinopathy.

According to the ROC analysis results, serum fractalkine levels showed moderate discriminative ability in distinguishing patients with and without retinopathy [AUC: 0.736 (95% CI: 0.634–0.838)]. At a cut-off value of 0.455 ng/mL, fractalkine was associated with 81.5% sensitivity and 56.3% specificity (positive predictive value: 86.3%, negative predictive value: 47.4%) (Figure 2).

**Figure 3:**
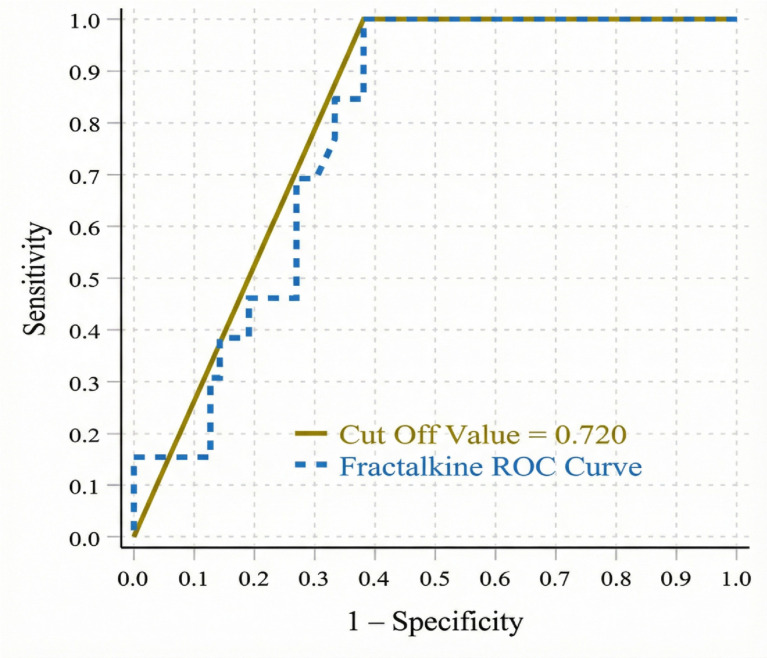
ROC curve of serum fractalkine levels for distinguishing the severe stage of non-proliferative diabetic retinopathy from the mild and moderate stages.

In the ROC analysis performed to assess disease severity in NPDR, serum fractalkine levels showed moderate discriminative ability in distinguishing the severe stage from the mild and moderate stages, with an AUC of 0.784 (95% CI: 0.679–0.888). At a cut-off value of 0.720 ng/mL, fractalkine was associated with 100% sensitivity and 61.9% specificity, with a positive predictive value of 35.1% and a negative predictive value of 100% (Figure 3).

## 4. Discussion

Diabetic retinopathy (DR) remains a common and vision-threatening microvascular complication of diabetes. Despite major advances in imaging and therapy, there is still a lack of reliable non-invasive biomarkers for early detection and risk stratification. Our findings, in this context, support growing evidence that circulating fractalkine (CX3CL1) may reflect underlying pathological processes across different stages of DR. Accordingly, the present study should be regarded as an early exploratory, hypothesis-generating investigation rather than a predictive or clinically applicable biomarker study.

In this study, individuals with DR had significantly higher serum fractalkine levels than those without retinopathy. Although higher concentrations were observed in patients with severe NPDR, serum fractalkine levels did not demonstrate a consistent linear correlation with established disease severity indices, and these findings should therefore be interpreted with caution. Experimental and clinical studies have shown that fractalkine participates in angiogenic and inflammatory pathways relevant to DR pathophysiology. You et al. reported elevated fractalkine expression in ocular tissues of patients with DR, supporting its involvement in retinal neovascular mechanisms, while Mills et al. showed that fractalkine-induced microglial activation disrupts retinal vasoregulation in early-stage disease. Together, these studies provide biological context for fractalkine involvement in DR without implying direct retinal specificity of circulating measurements [13,25]. In this context, the interpretation of circulating fractalkine levels should take into account that systemic inflammatory activity related to diabetes may contribute to the observed associations, independent of retinopathy-specific mechanisms. Unlike prior experimental and intraocular studies that primarily focused on local retinal or vitreous fractalkine activity, the present study extends existing evidence by providing clinical data based on circulating serum fractalkine levels, thereby offering a non-invasive and systemic perspective on inflammation in the context of DR.

In this framework, DR may be viewed as a clinically well-defined manifestation of generalized diabetic microangiopathy, and the observed associations with serum fractalkine are therefore more appropriately interpreted within a systemic microvascular and inflammatory context rather than as evidence of retina-specific pathology. Recent clinical evidence indicates that elevated circulating fractalkine levels are associated with endothelial dysfunction, atherosclerotic burden, and adverse cardiovascular outcomes, supporting its role as a marker of systemic vascular inflammation rather than organ-specific pathology [26].

Beyond fractalkine, other chemokines have also been implicated in inflammatory pathways relevant to DR progression. Monickaraj et al. identified CXCL1 as a mediator of leukocyte recruitment and blood–retinal barrier disruption in diabetic retinal tissue, highlighting the broader role of chemokine-regulated inflammation in microvascular injury [27]. In parallel, Mendiola et al. showed that the fractalkine–CX3CR1 axis modulates microglial activation and perivascular clustering in diabetic retinal models [15]. Collectively, these findings reinforce the biological plausibility of our results by positioning fractalkine within a broader chemokine network implicated in the pathogenesis of DR. Importantly, experimental studies have demonstrated that fractalkine signaling exerts context-dependent effects on microglial physiology, modulating the balance between proinflammatory activation and homeostatic regulation. In specific settings, preservation of CX3CL1–CX3CR1 signaling has been shown to attenuate excessive microglial activation and to support neuroinflammatory homeostasis, underscoring the complex and bidirectional nature of fractalkine biology [28].

In our cohort, no significant difference was observed between the NPDR and PDR groups. This finding may reflect interindividual variability in CX3CR1 receptor polymorphisms or variation in A Disintegrin and Metalloproteinase Domain-Containing Protein 10/17 (ADAM10/ADAM17) metalloproteinase activity, both of which can alter fractalkine processing and signaling. Previous vitreous-based studies, including those by Abu El-Asrar et al., have highlighted the interaction between fractalkine and VEGF in proliferative disease; however, serum measurements do not allow direct inference regarding intraocular chemokine dynamics [29]. Therefore, while our findings align with retinal mechanistic studies, further work incorporating ocular samples is warranted to clarify stage-specific differences.

We found that diabetes duration was significantly longer in patients with DR than in those without retinopathy. However, the duration was similar between the NPDR and PDR groups and across NPDR substages. This pattern suggests that DR progression is driven by additional factors beyond cumulative hyperglycemia exposure. This view is consistent with longitudinal cohort data, including The United Kingdom Prospective Diabetes Study 33 (UKPDS) and the Los Angeles Latino Eye Study, which show that although longer diabetes duration increases DR risk, it does not consistently predict severity [30,31]. These findings underscore the multifactorial nature of DR progression, involving metabolic, vascular and inflammatory pathways.

Glycemic control remains a major determinant of DR development, as demonstrated by landmark trials such as UKPDS 33 and the EDIC follow-up of the DCCT cohort [32,33]. The concept of metabolic memory, as demonstrated in the retrospective study by Varadhan et al., indicates that while early poor glycemic control increases the risk of retinopathy, subsequent tight glycemic control can still reduce this risk by up to 80% [34]. Consistent with these results, fasting glucose and HbA1c levels were higher in our DR group compared with individuals without retinopathy. However, no significant differences were observed between the NPDR and PDR groups or across NPDR severity stages. This suggests that once retinopathy has developed, glycemic exposure alone may not adequately account for differences in clinical severity. The concept of metabolic memory—defined by the lasting effects of earlier glycemic control—may help explain these findings and warrants further evaluation in prospective studies.

The HbA1c-stratified analyses indicated that the association between circulating fractalkine and DR became more evident among individuals with poorer glycemic control, suggesting that this relationship is influenced by the overall metabolic and inflammatory milieu rather than representing a glucose-independent or retina-specific effect.

This study found a significant association between DR and nephropathy, with higher levels of serum creatinine, urea, and microalbumin/creatinine ratio in patients with DR compared to those without. These results reinforce the link between diabetic microvascular complications. Previous studies, including those by El-Asrar AM et al., Li et al., Manavi, and Butt, have similarly demonstrated that the prevalence of both retinopathy and microalbuminuria increases with diabetes duration and they are positively correlated [35,36,37,38]. Notably, we observed a progressive increase in urine microalbumin levels from mild to severe NPDR, indicating worsening renal involvement with advancing retinopathy. However, no significant differences in renal parameters were noted between NPDR and PDR groups. These findings suggest that early and intensive glycemic control, especially in patients with mild NPDR, may help slow the progression of both retinopathy and nephropathy, supporting conclusions from Rasheed et al. in long-term diabetic patients [39]. Consistent with these findings, studies in diabetic kidney disease have demonstrated that activation of the CX3CL1–CX3CR1 axis reflects renal microvascular inflammation and leukocyte recruitment, with circulating fractalkine levels mirroring systemic inflammatory injury rather than kidney-specific mechanisms [40].

Multivariable logistic regression analyses identified serum fractalkine as an independent correlate of DR after adjustment for major clinical variables associated with retinopathy. While the cross-sectional nature of the study precludes causal inference, these findings suggest that systemic fractalkine levels are associated with DR-related inflammatory pathways beyond traditional risk factors.

ROC curve analyses demonstrated that serum fractalkine showed moderate discriminative ability between individuals with and without DR and was associated with severe NPDR. Subgroup analyses involving PDR and severe NPDR should be interpreted with caution, given the limited number of cases in these subgroups. However, these findings should be interpreted as exploratory and associative, as the cross-sectional design does not permit conclusions regarding diagnostic accuracy, prognostic value, or clinical utility. Accordingly, the proposed cut-off values should be regarded as descriptive findings rather than validated clinical thresholds. It should not be overlooked that prospective studies are required to determine whether serum fractalkine provides incremental value beyond established clinical markers. Notably, the high sensitivity values observed in certain ROC analyses should be interpreted with caution, as the limited number of patients in specific subgroups, particularly those with PDR and severe NPDR, may lead to overestimation or instability of sensitivity estimates. However, the observed AUC values indicate moderate discriminative ability and are inferior or comparable to established clinical predictors, rendering serum fractalkine insufficient for standalone diagnostic use.

Within contemporary DR screening frameworks that increasingly rely on automated and artificial intelligence–supported approaches, the integration of systemic information may help contextualize imaging-based findings [18,19]. In this regard, the present study demonstrates that serum fractalkine levels are associated with both the presence and severity of DR, suggesting a potential supportive role alongside established clinical and ophthalmologic assessments. Rather than functioning as a standalone diagnostic marker, circulating fractalkine may contribute additional insight into underlying inflammatory and microvascular activity that is not directly captured by retinal imaging alone. This complementary perspective may be particularly relevant for patients identified through assistive or remote screening pathways, in whom systemic risk profiling could inform the need for closer ophthalmologic surveillance or intensified metabolic management. Nevertheless, given the cross-sectional and exploratory nature of the study, these findings should be interpreted cautiously, particularly with respect to how systemic inflammatory parameters such as fractalkine may be incorporated into future assistive care strategies.

### Limitations

However, several limitations should be acknowledged. First, the absence of a healthy, non-diabetic control group limits the ability to determine whether elevated serum fractalkine levels are specific to DR or instead reflect systemic inflammation associated with diabetes itself. Consequently, diabetes-related inflammatory processes cannot be fully disentangled from retinopathy-specific alterations in the present study. Second, fractalkine was measured exclusively in serum, and no intraocular (vitreous or aqueous) measurements were available. Therefore, direct inference regarding local retinal or microglial mechanisms is limited, and the biological interpretation of these findings should be considered indirect. Third, although the overall sample size was adequate, the relatively small number of participants in certain subgroups, particularly those with PDR and severe NPDR, may affect the stability of multivariable regression models and the precision of ROC estimates. This limitation may reduce statistical power and limit the generalizability of these findings. Moreover, the relatively high number of predictors included in the multivariable logistic regression model in relation to the limited number of outcome events within these subgroups may have increased the risk of overfitting. In addition, residual confounding cannot be excluded, as detailed information on long-term glycemic exposure, specific antidiabetic treatment regimens, antihypertensive and lipid-lowering therapies (including statins), anti-inflammatory medication use, the duration and severity of diabetic nephropathy, and the presence of other microvascular complications such as diabetic neuropathy was not fully captured or adjusted for in the present analyses. Finally, we did not evaluate genetic or molecular determinants of the fractalkine–CX3CR1 axis, such as CX3CR1 polymorphisms or ADAM10/ADAM17 activity, and fractalkine was measured at a single time point, precluding assessment of temporal variability or longitudinal changes in relation to disease progression.

## 5. Conclusions

This study demonstrates a significant association between serum fractalkine levels and both the presence and severity of DR. Serum fractalkine levels were associated with DR and showed moderate discriminative ability, particularly in severe nonproliferative stages. However, given the cross-sectional design, these findings should be interpreted as associative rather than predictive. The observed associations may predominantly reflect systemic inflammatory processes associated with DR rather than direct evidence of local retinal or microglial mechanisms. Overall, serum fractalkine appears to be associated with the severity of diabetic microvascular disease, including retinopathy; however, its retinal specificity and incremental clinical value beyond established glycemic and renal markers remain unproven. Accordingly, the retinal specificity and clinical applicability of serum fractalkine cannot be established based on the present data, including its role within contemporary assistive or automated screening frameworks. Prospective longitudinal studies are required to elucidate temporal relationships, clarify biological relevance, and determine incremental value beyond established clinical markers.

## Figures and Tables

**Table 1:** Comparison of demographic and clinical variables between patients with and without diabetic retinopathy.

Parameters	Without Retinopathy (*n* = 32)	With Retinopathy (*n* = 108)	*p*
Age, year	53 (48–59)	59 (52–66)	0.071
DM duration, year	3.5 (1–30)	10 (1–35)	<0.001
Gender, *n* (%)			
Female	13 (40.6)	58 (53.7)	0.194
Male	19 (59.4)	50 (46.3)	
BMI, kg/m^2^	30.2 ± 4.9	29 ± 4.4	0.180
HT, *n* (%)			
Absent	26 (81.2)	68 (63)	0.053
Present	6 (18.8)	40 (37)	
Smoking Status, *n* (%)			
No	29 (90.6)	91 (84.3)	0.366
Yes	3 (9.4)	17 (15.7)	
Medications, *n* (%)			
Oral Antidiabetic	23 (71.9)	46 (42.6)	0.004
Insulin	2 (6.3)	7 (6.5)	0.963
Insulin + OAD	7 (21.9)	51 (42.2)	0.011
Antihypertensive	6 (18.8)	36 (33.3)	0.114

Data are presented as mean ± SD or median for non-normally distributed variables. Comparisons were made using the independent t-test for normal distribution, Mann–Whitney U test for non-normal distribution, and chi-square (χ^2^) test for categorical variables. Statistical significance was set at *p* < 0.05. DM: diabetes mellitus, OAD: oral antidiabetic drugs, BMI: body mass index, HT: hypertension.

**Table 2:** Comparison of serum fractalkine levels and laboratory values between patients with and without diabetic retinopathy.

Parameters	Without Retinopathy (*n =* 32)	With Retinopathy (*n* = 108)	*p*
Fractalkine, ng/mL	0.4 (0.4–0.95)	0.7 (0.42–0.85)	<0.001
CRP, mg/L	2.8 (1.5–5.5)	3.7 (2.1–7.3)	0.773
Glucose, mg/dL	152.5 (119–223)	212.5 (163–276)	0.022
HbA1c, %	7.6 (6.7–9.8)	9.1 (8.1–9.8)	0.004
HOMA-IR	4.7 (2.6–7.1)	4.4 (2.3–6.7)	0.749
Urea, mg/dL	29.9 (23–37)	34 (28–41)	0.008
UACR, mg/g	13.9 (7.5–58.2)	35.3 (16.6–157)	0.003
Creatinine, mg/dL	0.7 (0.6–0.9)	0.9 (0.7–1)	<0.001
LDL-c, mg/dL	117 (89–152)	117.5 (90–152)	0.656
HDL-c, mg/dL	44.5 (36–50)	42 (38–53)	0.356
TG, mg/dL	191.5 (120–242)	175.5 (126–232)	0.580
Total Cholesterol, mg/dL	184 (166–221)	194.5 (153–231)	0.962
WBC, 10^3^/mm^3^	7.3 (6.9–9.3)	8.2 (7.3–10.2)	0.013

Data are presented as mean ± SD or median for non-normally distributed variables. Comparisons were made using the independent t-test for normal distribution, Mann–Whitney U test for non-normal distribution. Statistical significance was set at *p* < 0.05. CRP: c-reactive protein, HbA1c: hemoglobin A1c, HOMA-IR: homeostasis model assessment of insulin resistance, UACR: urine albumin-to-creatinine ratio, LDL-c: low-density lipoprotein, HDL-c: high-density lipoprotein, TG: triglyceride, WBC: white blood cell count.

**Table 3:** Univariate and Multivariate Logistic Regression Analysis of Variables Associated with Diabetic Retinopathy.

	Univariate Model			Multivariate Model		
	OR	%95 CI	*p*	OR	%95 CI	*p*
Duration of diabetes	1.18	1.09–1.28	<0.001	1.17	1.06–1.28	0.001
Fractalkine	25.3	4.1–154.4	<0.001	10.2	1.2–89.6	0.036
Glucose	1.01	1.00–1.01	0.032			
HbA1c	1.42	1.11–1.81	0.005	1.42	1.08–1.87	0.012
UACR	**1.001**	**1–1.001**	**0.201**			
Creatinine	22.75	3.41–152.0	0.001	20.78	1.97–219.1	0.012
WBC	1.32	1.06–1.65	0.014			

Logistic regression was performed using the Forward LR (Likelihood Ratio) method. The odds ratio (OR) and 95% confidence interval (CI) are provided for each variable along with *p*-values. Statistically significant *p*-values (<0.05) are bolded. HbA1c: hemoglobin A1c; UACR: urine albumin-to-creatinine ratio; WBC: white blood cell count.

**Table 4:** HbA1c-Stratified Multivariable Logistic Regression Analysis for Diabetic Retinopathy in Individuals with HbA1c < 7%.

	Univariate Model			Multivariate Model		
	OR	%95 CI	*p*	OR	%95 CI	*p*
Duration of diabetes	1.25	1.03–1.53	0.023	1.22	0.97–1.52	0.077
Fractalkine	7.53	0.52–108	0.138			
Creatinine	2.40	1.59–3.62	0.032	2.21	0.59–8.20	0.074
UACR	1.01	0.99–1.19	0.325			

Logistic regression was performed using the Forward LR (Likelihood Ratio) method. The odds ratio (OR) and 95% confidence interval (CI) are provided for each variable along with *p*-values. UACR: urine albumin-to-creatinine ratio.

**Table 5:** HbA1c-Stratified Multivariable Logistic Regression Analysis for Diabetic Retinopathy in Individuals with HbA1c ≥ 7%.

	Univariate Model			Multivariate Model		
	OR	%95 CI	*p*	OR	%95 CI	*p*
Duration of diabetes	1.14	1.04–1.25	0.003	1.15	1.04–1.27	0.006
Fractalkine	34.0	3.38–342	0.003	52.6	3.35–825	0.005
Creatinine	1.25	1.00–11.0	0.023	0.81	0.08–7.9	0.075
UACR	1.00	1.00–1.01	0.386			

Logistic regression was performed using the Forward LR (Likelihood Ratio) method. The odds ratio (OR) and 95% confidence interval (CI) are provided for each variable along with *p*-values. UACR: urine albumin-to-creatinine ratio.

**Table 6:** Comparison of serum fractalkine levels and laboratory parameters between patients with proliferative and non-proliferative diabetic retinopathy.

Parameters	Proliferative DR (*n* = 32)	Non-Proliferative DR (*n* = 76)	*p*
Fractalkine, ng/mL	0.5 (0.38–1.03)	0.7 (0.44–0.83)	0.274
CRP, mg/L	4.5 (2–7)	3.1 (1–6)	0.668
Glucose, mg/dL	209 (165–300)	215.5 (158–265)	0.696
HbA1c, %	9.6 (8–9.8)	9 (8.4–9.7)	0.279
HOMA-IR	3.7 (2.3–6.3)	4.8 (2.3–7.2)	0.623
Urea, mg/dL	33 (31–54)	35 (26–41)	0.497
UACR, mg/g	65.6 (14–150)	33.3 (16–244)	0.218
Creatinine, mg/dL	0.8 (0.8–1)	0.9 (0.7–1)	0.893
LDL-c, mg/dL	127 (90–150)	118 (90–154)	0.326
HDL-c, mg/dL	44 (38–53)	43 (38–54)	0.731
TG, mg/dL	187.8 ± 94.5	181.8 ± 87	0.751
Total Cholesterol, mg/dL	204 (147–231)	190 (153–231)	0.214
WBC, 10^3^/mm^3^	8.2 (7.4–9.7)	8.2 (7.2–10.5)	0.797

*p*-values were calculated using the independent samples t-test for variables with normal distribution, and the Mann–Whitney U test for variables with non-normal distribution. Normality was assessed using the Shapiro–Wilk test. DR: diabetic retinopathy, CRP: c-reactive protein, HbA1c: hemoglobin A1c, HOMA-IR: homeostasis model assessment of insulin resistance, UACR: urine albumin-to-creatinine ratio, LDL-c: low-density lipoprotein, HDL-c: high-density lipoprotein, TG: triglyceride, WBC: white blood cell count.

**Table 7:** Comparison of serum fractalkine levels and laboratory parameters in non-proliferative DR.

Parameters	Mild Non-Proliferative DR (*n =* 23)	Moderate Non-Proliferative DR (*n =* 40)	Severe Non-Proliferative DR (*n =* 13)	*p*
Fractalkine, ng/mL	0.68 (0.41–0.79)	0.63 (0.46–0.91)	0.83 (0.45–0.93) *	0.004
CRP, mg/L	2.2 (1–5)	4.4 (2–13)	2.5 (2–8)	0.092
Glucose, mg/dL	218 (165–269)	197.5 (136–267)	244 (162–246)	0.992
HbA1c, %	9.2 (7.9–10.1)	8.8 (8–9.4)	9.2 (8.7–9.7)	0.788
HOMA-IR	5 (3.3–6.7)	4.7 (2–7.6)	4.1 (2.4–7)	0.881
Urea, mg/dL	33 (25–36)	33.5 (24–41)	36.7 (28–41)	0.777
UACR, mg/g	20.8 (8.7–119) ^¥^	49.3 (25–325)	45 (30–338)	0.015
Creatinine, mg/dL	0.8 (0.7–0.9)	0.9 (0.7–1.3)	0.9 (0.7–1.2)	0.589
LDL-c, mg/dL	115.5 ± 36.5	118.7 ± 43.5	125.9 ± 33.7	0.754
HDL-c, mg/dL	41 (36–55)	40 (38–53)	43 (40–54)	0.328
TG, mg/dL	164.8 ± 67.7	193.5 ± 100.7	176 ± 69.7	0.442
Total Cholesterol, mg/dL	183 (169–233)	190 (147–212)	217 (181–251)	0.251
WBC, 10^3^/mm^3^	8.5 ± 2.5	8.7 ± 2.1	8.7 ± 1.9	0.772

*p*-values were calculated using the Kruskal–Wallis (K) test for non-normally distributed variables and the ANOVA (A) test for normally distributed variables. Statistical significance was set at *p* < 0.05. * Significant difference compared to the moderate non-proliferative group (*p* < 0.05). ^¥^ Significant difference compared to the severe non-proliferative group (*p* < 0.05). DR: diabetic retinopathy, CRP: c-reactive protein, HbA1c: hemoglobin A1c, HOMA-IR: homeostasis model assessment of insulin resistance, UACR: urine albumin-to-creatinine ratio, LDL-c: low-density lipoprotein, HDL-c: high-density lipoprotein, TG: triglyceride, WBC: white blood cell count.

## Data Availability

The data that support the findings of this study are available from the corresponding author upon reasonable request.

## Supplementary Materials

The following supporting information can be downloaded at: https://www.mdpi.com/article/10.3390/medicina62020312/s1: The completed STROBE checklist.

## Abbreviations

DR	Diabetic retinopathy
PDR	Proliferative diabetic retinopathy
NPDR	Non-proliferative diabetic retinopathy
VEGF	Vascular endothelial growth factor
CX3CL1	Fractalkine
CX3CR1	CX3C chemokine receptor 1
T2D	Type 2 diabetes
ADA	American diabetes association
ICDR	International clinical diabetic retinopathy severity scale
ETDRS	Early treatment diabetic retinopathy study
ELISA	Enzyme-linked immunosorbent assay
BMI	Body mass index
HbA1c	Hemoglobin A1c (Glycated Hemoglobin)
CRP	C-Reactive Protein
ADAM10	A Disintegrin and Metalloproteinase Domain-Containing Protein 10
ADAM17	A Disintegrin and Metalloproteinase Domain-Containing Protein 17
UKPDS 33	The United Kingdom Prospective Diabetes Study 33
ROC	Receiver Operating Characteristic
AUC	Area under the curve
TMB	Tetramethylbenzidine
